# Astrocytes and Microglia as Major Players of Myelin Production in Normal and Pathological Conditions

**DOI:** 10.3389/fncel.2020.00079

**Published:** 2020-04-07

**Authors:** Elisabeth Traiffort, Abdelmoumen Kassoussi, Amina Zahaf, Yousra Laouarem

**Affiliations:** U1195 Inserm, University Paris-Saclay, Kremlin-Bicêtre, France

**Keywords:** oligodendrocyte, myelination, remyelination, astrocyte, microglia

## Abstract

Myelination is an essential process that consists of the ensheathment of axons by myelin. In the central nervous system (CNS), myelin is synthesized by oligodendrocytes. The proliferation, migration, and differentiation of oligodendrocyte precursor cells constitute a prerequisite before mature oligodendrocytes extend their processes around the axons and progressively generate a multilamellar lipidic sheath. Although myelination is predominately driven by oligodendrocytes, the other glial cells including astrocytes and microglia, also contribute to this process. The present review is an update of the most recent emerging mechanisms involving astrocyte and microglia in myelin production. The contribution of these cells will be first described during developmental myelination that occurs in the early postnatal period and is critical for the proper development of cognition and behavior. Then, we will report the novel findings regarding the beneficial or deleterious effects of astroglia and microglia, which respectively promote or impair the endogenous capacity of oligodendrocyte progenitor cells (OPCs) to induce spontaneous remyelination after myelin loss. Acute delineation of astrocyte and microglia activities and cross-talk should uncover the way towards novel therapeutic perspectives aimed at recovering proper myelination during development or at breaking down the barriers impeding the regeneration of the damaged myelin that occurs in CNS demyelinating diseases.

During the last decades, the production of myelin in the central nervous system (CNS) has been the focus of a multitude of studies in the context of development or regeneration. Oligodendrocytes, the cells synthesizing myelin in the CNS, were initially found in hind-jawed fishes (Zalc and Colman, 2000; Hartline and Colman, 2007; Zalc, 2016). These cells derive from oligodendrocyte progenitor cells (OPCs) occurring in several waves. In mice, the first wave arises at embryonic day (E) 12.5 in the ventral domain of the ventricular wall in the developing brain and spinal cord. The second one emerges more dorsally at E15.5 in both regions and the last one arises specifically in the perinatal cortex around birth (Pringle and Richardson, 1993; Timsit et al., 1995; Trousse et al., 1995; Spassky et al., 1998; Olivier et al., 2001). Unexpectedly, the destruction of one of these waves in the telencephalon induces the remaining cells to compensate for the lost oligodendrocytes thus suggesting functional similarity. In agreement with this observation, ventrally derived oligodendroglial cells strongly decline early after birth in the postnatal dorsal forebrain and are replaced by dorsally derived cells, which thus constitute more than 80% of oligodendroglial cells present in the adult corpus callosum and cerebral cortex (Kessaris et al., 2006; Tripathi et al., 2011). Myelination itself progresses according to typical sequences, which are spatially and temporally determined. In humans, after 16 weeks of gestation, myelin can be detected in the fasciculus cuneatus, and shortly after occurs in the cerebellar and pyramidal tracts. Then, myelination proceeds rapidly during the first year from the occipital to the fronto-temporal lobes. Regions devoted to basic homeostasis are proposed to be myelinated before areas involved in more intricate tasks like the frontal cortex. Late myelinated areas are typically myelinated at a lower level than early myelinating regions (Brody et al., 1987; Kinney et al., 1988). More recent data derived from serial reconstructions of the adult mouse cortex showed that the degree of myelination can even vary along a single axon. Thus, neurons located in superficial cortical layers exhibit both myelinated- and large unmyelinated segments, contrasting with the pattern observed for instance in the spinal cord or the optic nerve where myelinated axons display regular internodes (Tomassy et al., 2014).

The finding that the CNS can regenerate myelin lost upon various types of insults was observed much earlier than the characterization of oligodendrocyte and myelin generation. Indeed, almost 60 years ago, occasional myelin sheaths were visualized a short time after demyelination was induced into the subpial cord by repeated withdrawal and reinjection of cerebrospinal fluid (Bunge et al., 1961). However, the demonstration that central remyelination was able to restore secure conduction in the demyelinated spinal cord and to lead to functional recovery was provided only two decades later (Smith et al., 1979). Since then, thousands of publications were dedicated to this process notably in the aim to investigate how new OPCs are recruited to the demyelinating area and differentiate into myelinating oligodendrocytes (Gallo and Armstrong, 2008; Clemente et al., 2013; Domingues et al., 2016; Lloyd et al., 2017).

Although in both development and repair, the oligodendrocyte is the pivotal cell type for myelin production, its function is widely influenced by the other glial cells, namely astrocytes and microglia. This review is aimed at revisiting the present knowledge regarding communication between oligodendroglial, astroglial and microglial cells in the context of myelin development and regeneration with a specific focus on most recent advances.

## Astrocyte-Oligodendrocyte Communication in Developmental Myelin Production

In the mouse spinal cord and cerebral cortex, the first mature astrocytes expressing the glial fibrillary acidic protein (GFAP) can be detected at the embryonic day (E) 12.5 and 16.5 respectively, thus before the start of OPC maturation and axon myelination taking place during the first three postnatal weeks (Miller et al., 1985; Qian et al., 2000). Like neurons and oligodendrocytes, astrocytes are derived from the neuroepithelium from which they migrate throughout the CNS along radial glia processes (Molofsky and Deneen, 2015).

### Astrocyte-Derived Trophic Factors and Cytokines Control Oligodendrogenesis

The crucial role of astrocytes was initially related to the finding that these cells are the main producers of the platelet-derived growth factor (PDGF). Acting through its receptor PDGFRa, PDGF promotes OPC proliferation and mobility and, also inhibits OPC differentiation towards mature oligodendrocytes, by generating a graded response requiring activation of the phosphoinositide 3-kinase and/or phospholipase C γ pathways. Astrocyte-derived PDGF was thus proposed to drive the clock that times oligodendrocyte development (Raff et al., 1988; Richardson et al., 1988; McKinnon et al., 2005). Besides PDGF, astrocytes secrete other growth factors. Brain-derived neurotrophic factor (BDNF) is namely required to potentiate myelination during the early postnatal development as indicated by BDNF knockout heterozygous mice, which exhibit delayed CNS myelination (Cellerino et al., 1997). In the same line, under stress conditions such as those occurring during prolonged cerebral hypoperfusion at birth, astrocyte-derived BDNF also supports OPC maturation in a TrkB-dependent manner as shown *in vitro* in sublethal CoCl2 exposition of OPC / astrocyte primary co-cultures and, *in vivo*, in a transgenic mouse line in which BDNF expression was specifically downregulated in astrocytes (Miyamoto et al., 2015). The observation that ciliary neurotrophic factor (CNTF) synthesis by astrocytes starts at the onset of myelination in the rodent optic nerve (Stöckli et al., 1991; Dobrea et al., 1992) led to suggest its physiological role on myelination, as well. The strong pro-myelinating effects of CNTF and other members of the family such as the leukemia inhibitory factor (LIF) in myelinating co-cultures further supported this hypothesis. CNTF acts on oligodendrocytes by favoring their final maturation *via* a mechanism involving the 130 kDa glycoprotein receptor common to the CNTF family and transduced through the Janus kinase (JAK) pathway (Stankoff et al., 2002). In contrast, other astrocyte-derived secreted proteins negatively regulate oligodendrocyte biology. For instance, the chemokine CXCL1 transiently expressed at a high level during spinal cord development signals through the chemokine receptor CXCR2 expressed by immature OPCs. Migrating OPCs were proposed to enter the presumptive white matter where they may encounter an environment in which astrocytes transiently and locally express high CXCL1 levels. Through CXCR2, CXCL1 may inhibit PDGF-stimulated OPC migration before subsequently increasing OPC proliferation in concert with PDGF (Tsai et al., 2002).

### Astrocytes Control the Availability of Cues Regulating Oligodendrocyte Production

**Figure 1:**
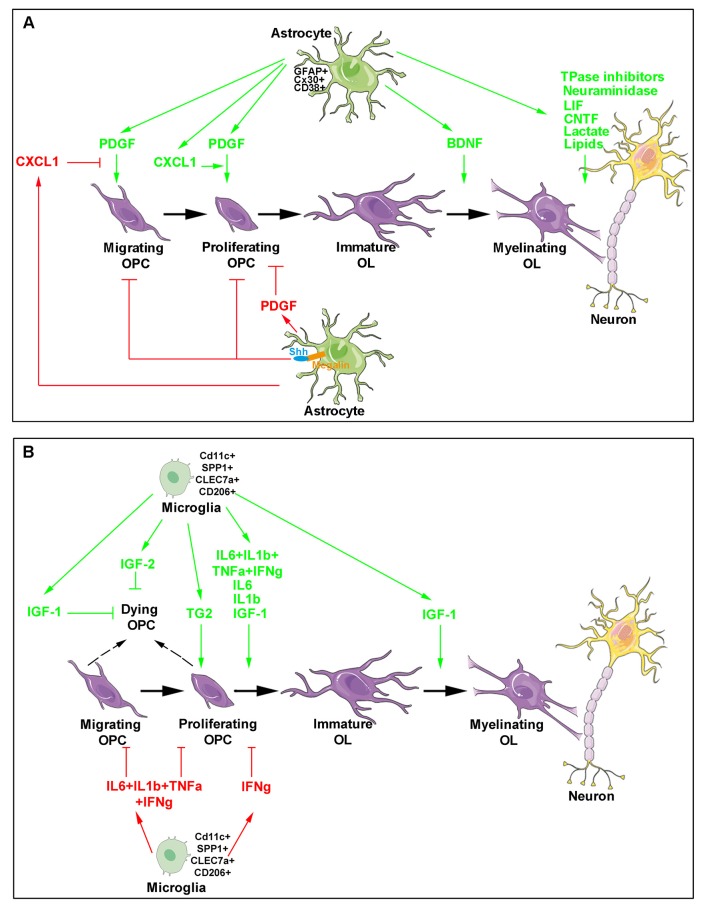
Major roles of astrocytes and microglia during developmental oligodendrogenesis and myelination. The different steps of oligodendroglial cell production (purple) are shown and include oligodendrocyte progenitor cell (OPC) migration, proliferation, differentiation as well as the maturation of immature oligodendrocytes into cells able to myelinate neuronal axons. Astrocyte- **(A)** and microglia- **(B)** derived molecules are indicated. The green arrows indicate molecules exhibiting a positive activity on oligodendrocyte lineage and myelin production (top in each panel) whereas the red arrows and blocking symbols indicate molecules displaying an inhibiting activity on oligodendroglial lineage production (bottom in each panel).

Besides the secretion of trophic factors, astrocytes are also able to control the bioavailability of oligodendrocyte-regulating cues (Figure 1A). The best-documented example was provided by the capacity of astrocytes to control the concentration of the morphogen Sonic Hedgehog (Shh) during OPC production in the optic nerve. Shh participates in the proliferation and migration of OPCs during the colonization of this nerve (Merchán et al., 2007). The multiligand receptor megalin, a member of the low-density lipoprotein receptor family able to bind Shh, was found to be exclusively expressed by astrocytes according to a dynamic pattern paralleling optic nerve colonization by OPCs arising from the optic chiasm and migrating to the retina. Indeed, when OPCs start their migration throughout the nerve at E14.5 in the mouse, megalin is more widely distributed in the region close to the optic chiasm whereas the distribution is reversed at E16.5 when the first OPCs reach the retina. Since, thereafter, megalin was found to be weakly and uniformly expressed all along the nerve, this receptor was proposed to control Shh internalization and its subsequent release at the suitable concentration during the various steps leading to oligodendrogenesis (Ortega et al., 2012).

### Astrocyte Integrity Is Required for Proper Myelination

Consistent with the above findings, the requirement of astrocyte integrity for the normal development of oligodendrocytes was shown by the analysis of transgenic mouse strains devoid of GFAP expression. Although viable, GFAP knockout mice exhibit abnormal myelination including the presence of actively myelinating oligodendrocytes in adults, nonmyelinated axons in the optic nerve, reduced myelin thickness in spinal cord and ultrastructural defects such as loosening of myelin sheaths (Liedtke et al., 1996). More recently, the invalidation of the transmembrane protein CD38, which possess ADP-ribosyl cyclase activity and is highly expressed in astrocytes, was similarly reported to result in altered astrocyte maturation and delayed oligodendrocyte differentiation during the postnatal development (Hattori et al., 2017). Furthermore, physical interactions between oligodendrocytes and astrocytes appear to be also required for myelination as shown by analysis of the role of connexins (Cx) 30 and Cx43 present on astrocytes and forming gap junctions with Cx32 and Cx47 present on oligodendrocytes. First, Cx47 mutants that cause the Pelizaeus–Merzbacher-like disease do not efficiently form functional channels with Cx43 (Orthmann-Murphy et al., 2007). Second, the deletion of both Cx47 and Cx30 in mice induces severe myelination defects including vacuole formation and thin myelin sheaths as well as a decreased number of oligodendrocytes (Tress et al., 2012). A mechanism possibly involved is proposed to be disruption of the metabolic support provided by astrocytes to oligodendrocytes in agreement with the gap junction-mediated unidirectional flux that transports the molecules preferentially from astrocytes to oligodendrocytes (Robinson et al., 1993).

### Astrocytes Are Indispensable for Axon Myelination

Accurate delineation of the molecular mechanisms supporting the role of astrocytes in myelination itself has never ceased for the last decades. Among the most relevant findings, the requirement of astrocytes for alignment and adherence of oligodendrocyte processes to axons was shown by using cocultures of retinal ganglion cells and optic nerve oligodendrocytes that led to propose a mechanism implicating neuraminidase-mediated removal of polysialic acid (PSA) from both cell types (Meyer-Franke et al., 1999). In agreement with this result, the abolition of the postnatal downregulation of PSA in oligodendrocytes from transgenic mice expressing the polysialyltransferase ST8SiaIV under the control of the proteolipid protein promoter indicated that PSA downregulation is required for efficient differentiation of oligodendrocytes, as well as for myelin formation and maintenance. However, *in vivo* implication of astrocytes is still lacking (Fewou et al., 2007). Another link between astrocytes and myelination was established by the demonstration that electrical activity in pre-myelinated axons increases myelination after OPCs mature to a promyelinating stage *via* the activity-dependent release of ATP from axons. ATP acts in a paracrine manner on astrocytes *via* the purinergic P2 receptor and induces the release of the LIF cytokine that ultimately stimulates myelination (Ishibashi et al., 2006). A specific myelinating CNS coculture system provided evidence that astrocytes are implicated rather in the promotion of rapid myelin growth than in initiation of myelination (Watkins et al., 2008).

### Astrocytes Provide Lipids for Myelin Sheath Production

Still consistent with their implication in myelination, astrocytes were also proposed to supply lactate to oligodendrocytes. Lactate constitutes a source of energy and a precursor of lipid synthesis including cholesterol altogether necessary for myelin production. Although astrocytes and oligodendrocytes are both in direct contact with blood vessels and may thus take up lactate from blood, the hypothesis is that lactate released from astrocytes through the monocarboxylate transporter (MCT) 4 may be supplied to oligodendrocytes possibly importing the molecule through MCT1 (Sánchez-Abarca et al., 2001; Rinholm et al., 2011). The requirement for local synthesis of cholesterol is consistent with the fact that myelin comprises about 80% of the brain cholesterol content and that peripheral cholesterol entry into the brain is largely precluded by the blood-brain barrier. During postnatal myelination, *de novo* synthesis of cholesterol is carried out by both astrocytes and oligodendrocytes. Astrocyte-derived cholesterol is then distributed to oligodendrocytes *via* lipoproteins contained in apolipoproteins, mostly the apolipoprotein E (ApoE) in the CNS, and secreted *via* the ATP-binding cassette transporter ABCA1 (Saher and Stumpf, 2015). Astrocytes provide a substantial fraction of the lipids incorporated into CNS myelin and in the absence of astrocyte lipid synthesis, oligodendrocytes are unable to finalize CNS myelination, leading to hypomyelinated and slower-conducting fibers in adulthood. Indeed, the specific inactivation of an essential co-activator of the transcription factor SREBP, the SREB cleavage activating protein (SCAP) in oligodendrocytes, resulted in lipid biosynthesis and myelination delay nevertheless recovering in adulthood. In contrast, when the enzyme was deleted in astrocytes or both astrocytes and oligodendrocytes, a persistent hypomyelination was observed. Thus, extracellular lipids seem to be supplied by astrocytes under conditions of compromised oligodendrocyte lipid synthesis. Moreover, full myelin synthesis requires an astrocyte lipid supply in addition to endogenous oligodendrocyte lipid synthesis (Camargo et al., 2017).

Besides providing lipids for the synthesis of myelin sheaths during development, astrocytes, namely those contacting the nodes of Ranvier, were also reported to reversibly modify myelin thickness and nodal gap length thus appropriately regulating conduction velocity in individual axons. In support of this observation, the reduction of exocytosis induced in transgenic mice expressing a dominant-negative fragment of the vesicle-associated membrane protein 2 (VAMP2) in astrocytes, exhibited detachment of adjacent paranodal loops of myelin from the axon, increased nodal gap length, thinning of myelin sheath in the optic nerve and finally decrease in visual acuity. These data led to propose that thrombin-dependent proteolysis of the cell adhesion molecule neurofascin 155 that attaches myelin to the axon, is inhibited by the vesicular release of thrombin protease inhibitors from perinodal astrocytes, which likely involves these cells in myelin remodeling necessary for optimal electrical conduction (Dutta et al., 2018).

The last evidence of the remarkable and specific relationship between astrocytes and myelination is finally provided in a model of neuromyelitis optica, a chronic inflammatory demyelinating disease characterized by the destruction of astrocytes and their foot processes in early lesions. Indeed, in a model of this disease, the death of oligodendrocytes was found to occur before granulocyte or macrophage/microglia infiltration, but only a few hours after the death of astrocytes induced in a complement-dependent manner (Wrzos et al., 2014).

## Microglia-Oligodendrocyte Communication in Developmental Myelin Production

Considered as resident myeloid cells of the CNS, microglia have been only recently characterized as the progeny of yolk sac-derived macrophages that start to enter the CNS at E9 through the blood vasculature before closure of the blood-brain barrier that occurs by E13 in mice (Ginhoux et al., 2010; Schulz et al., 2012; Kierdorf et al., 2013). Thereafter, at least under non-pathological conditions, microglia cells are autonomously maintained through proliferation (Askew et al., 2017). Microglia is crucial during neurodevelopment namely *via* its interaction with neuronal cells for wiring and neural circuit regulation (Thion et al., 2018).

### Distinct Patterns of Secreted Molecules for Microglia and Astrocyte-Mediated Control of OPC Generation

The first arguments supporting a role for microglia in developmental myelination were mainly derived from microglia-oligodendrocyte cocultures. They showed that microglia was able to stimulate the synthesis of sulfatide, a myelin-specific galactolipid, and to increase myelin-specific proteins, MBP and PLP in oligodendrocytes (Hamilton and Rome, 1994). Microglia activity was mimicked by conditioned medium derived from microglia cultures suggesting that the cells acted *via* the secretion of appropriate molecules. Together with promoting cell maturation, microglia were found to prevent OPC apoptosis by upregulating the nuclear factor-kappa B (NF-κB) p65 subunit *via* the recruitment of PI-3 kinase to the PDGFRa thus leading to a synergism with PDGF (Nicholas et al., 2001).

Although both microglia- and astrocyte-conditioned media were able to prevent OPC degeneration induced by growth factor deprivation in the culture medium during a short time, microglia-derived medium was unable to support OPC survival in the long-term compared to astrocytes. These differential effects were supported by distinct patterns of cytokine/growth factors correlated with differentially activated intracellular signaling pathways in OPCs (Pang et al., 2013). More specifically, mechanisms underlying enhanced microglia-induced oligodendrocyte differentiation were proposed to be related to IGF-1. IGF-1 levels were more than six-fold higher in microglia- than in astrocyte-derived medium and its expression was consistently detected in amoeboid microglial cells located in the corpus callosum until the seventh postnatal day, which corresponds to the time of morphological change of microglia towards the ramified phenotype present in adulthood unless microglia become activated. Consistently, exposure of microglia cultures to lipopolysaccharide leading to microglial activation, increased IGF-1 secretion (Kaur et al., 2006) and IGF-1 knockout mice display reduced oligodendrocyte survival, differentiation, and maturation (Ye et al., 2002). Moreover, IGF-2 also derived from microglia was found to prevent the TNFα-induced death of mature oligodendrocytes *in vitro* (Nicholas et al., 2002).

### Unique Phenotype of Neonatal Microglia Implicated in Developmental Myelination

As soon as the end of the 1970s, the existence of a specific subpopulation of microglial cells had been proposed in the early postnatal corpus callosum (Imamoto and Leblond, 1978). Unexpectedly, the pharmacological suppression of microglia activation by using the anti-inflammatory drug minocycline was reported to significantly inhibit oligodendrogenesis both *in vitro* in neurosphere culture and *in vivo* in the subventricular zone (SVZ) of the dorsal forebrain. This inhibition was mediated by the blockade of several proinflammatory cytokines including IL-1β, IL-6, TNF-α, and IFN-γ. Also, the observation that activated microglia significantly increased O4^+^ cells but decreased PDGFRa^+^ OPCs (Figure 1B) again confirmed microglia activity on oligodendrocyte maturation (Shigemoto-Mogami et al., 2014).

More recent data provided by two independent groups led to a major advance in the understanding of the identity and role of this microglial subpopulation that appeared to highly proliferate and display a rhomboid shape in contrast to microglia detected at the same time in the neighboring cortex and exhibiting small processes comparable to adult microglia (Hagemeyer et al., 2017; Wlodarczyk et al., 2017). Presently, neonatal microglia implicated in developmental oligodendrogenesis appear as a cell population with a dramatically different gene expression profile compared to the one from adult healthy mice. Based on the observation that during neuroinflammation, microglia expressing the integrin complement receptor CD11c are a major source of IGF-1, Wlodarczyk and collaborators detected a substantial increase of CD11c^+^ microglia during the first days after birth before a sharp decrease mainly in highly myelinating areas such as the developing corpus callosum and cerebellum. IGF-1 depletion in this subset led to a reduction in brain weight, a decrease in the expression of myelin proteins including PLP, MBP, MAG and MOG, and was finally associated with higher frequency of less myelinated fibers in the corpus callosum. Thus, as the major source of IGF-1, the CD11c^+^ microglia subset was proposed to play a critical role in primary myelination and neuronal support in the neonatal CNS (Wlodarczyk et al., 2017). Interestingly, the comparison of transcriptomes from sorted CD11c^+^ and CD11c^−^ microglia revealed that neonatal microglia, naive adult microglia and microglia derived from the experimental autoimmune encephalomyelitis (EAE) model of demyelination formed distinct global expression clusters. Unexpectedly, CD11c^+^ and CD11c^−^ subpopulations were relatively close together indicating that the major difference was related to developmental age rather than subpopulation phenotype. The identification of gene expression profiles associated with microglia from the different conditions showed that under demyelinating conditions, microglia cells were enriched for immune system genes consistent with their activated state. On the contrary, in the neonatal brain, microglia gene profile included genes involved in nervous system development displaying a neurogenic phenotype. Remarkably, upon a quite total ablation of microglia in the adult brain by using an inducible mouse strain, clusters of highly proliferating microglia were detectable throughout the CNS. Although expressing CD11c and nestin, the proliferating cells did not display a neurogenic gene expression profile confirming that the myelinogenic CD11c^+^ subset of microglia is unique to neonatal CNS (Wlodarczyk et al., 2017).

Independently, Hagemeyer and collaborators similarly identified this amoeboid microglia subset that they called “the fountain of microglia” specifically located in myelinating regions from postnatal day (P)1 to P8, displaying a high expression of the activation marker Mac3 and dramatically collapsing at P9. Microglia found in the postnatal subcortical (with an amoeboid shape) and cortical regions (with multiple cellular processes) were found to share their early origin from the same CX3CR1^+^ CNS endogenous precursors without any contribution from circulating blood monocytes. The pharmacological blockade of the receptor for the secreted cytokine, colony-stimulating factor 1 (CSF1), known to efficiently deplete microglia (Elmore et al., 2014), showed that early postnatal microglia is required for the proper induction of oligodendrocyte progenitors and subsequent myelination. Transcriptomic analysis of the studied subpopulation revealed genes also detected in disease models such as CLEC7a, SPP1, IGF-1, ANXA5, ITGAX, and GPNMB. An additional RNA sequencing analysis led to identify change in gene expression profile between P7 and P10, the former being related to phagocytosis, migration, priming of microglia more particularly described during aging and disease while the latter was associated with apoptosis and necrosis according to a profile further increased in adulthood. Thus, a specificity of neonatal microglia in P7 corpus callosum was the expression of SPP1, CLEC7A, and CD206 since none of them could be detected in cortical microglia (Staszewski and Hagemeyer, 2019). Importantly, functional microglia are also required for OPC homeostasis in the adult brain in agreement with the severe white matter abnormalities observed in patients suffering from microgliopathy (Hagemeyer et al., 2017).

### The Extracellular Matrix at the Crossroads of Microglia and OPC Activity

The investigation of how OPCs integrate signals from the matrix and other glial cells led to a growing interest for the adhesion G protein-coupled receptor (aGPCR) family, the second-largest class of GPCRs playing crucial roles in developmental processes (Mehta and Piao, 2017). The finding that the genetic loss of one of its members, GPR56, was phenocopied by the specific deletion of the enzyme microglia-derived transglutaminase 2 (TG2) in microglia both resulting in the decrease of OPC cell division and number of mature oligodendrocytes, as well as to hypomyelination during postnatal CNS development, led to identify GPR56 as the receptor of TG2. In agreement with the ability of TG2 to bind ECM proteins to exert its crosslinking activity, the activation of GPR56 by TG2 was found to require the ECM protein laminin to promote OPC proliferation according to a mechanism leading to the dissociation of GPR56 N- and C-terminal fragments allowing the agonist to initiate G-protein signaling and subsequently RhoA activation. Thus, microglia TG2, extracellular laminin, and OPC GPR56 provide additional evidence of the critical role of microglia/oligodendrocyte communication for developmental oligodendrogenesis (Giera et al., 2015). Remarkably, laminin is also involved in OPC proliferation by promoting the metalloproteinase-mediated cleavage of dystroglycan, one of its OPC surface receptors (Leiton et al., 2015). Moreover, other ECM glycoproteins play a crucial role in myelination as, for instance, anosmin-1 characterized as an important modulator of oligodendrocyte progenitor lineage progression likely *via* FGFR1/ERK1/2 signaling activation, which results in the control of MBP expression, myelin formation and conduction velocity (Murcia-Belmonte et al., 2016). The enhanced differentiation of induced pluripotent stem cells into myelin-expressing oligodendrocytes promoted by the functionalization of culture substrates using brain ECM prepared from decellularized human brain tissue further supports the critical activity of ECM in myelination (Cho et al., 2019).

## Influence of Astrocytes During Remyelination

### Reactive Astrocytes Provide Pro-regenerative Trophic Factors

As in other diseases, astrocytes become reactive after CNS demyelination. Astrocyte reactivity includes hypertrophy of the cells and upregulation of intermediate filament proteins such as GFAP and vimentin. The requirement of astrocytes for oligodendrocyte-mediated remyelination has been established more than 15 years ago (Blakemore et al., 2003; Talbott et al., 2005) and more recently reported to depend on the presence of androgen hormones in male mice (Bielecki et al., 2016). The delay in astrocyte activation in mice lacking IL-1β and the finding that activated microglia release Il-1β, were the first arguments supporting the idea that microglia activation is a pre-requisite for astrocyte activation. In a consistent manner, microglia-derived IL-1β is able to upregulate CNTF. Added to astrocyte cultures, CNTF induces astrocyte activation (Herx et al., 2000; Albrecht et al., 2003), which promotes fibre myelination *in vitro* (Nash et al., 2011). *In vivo*, astrocytes derived from mice infected with the mouse hepatitis virus, a model for multiple sclerosis (MS), secrete high levels of CNTF during the remyelination phase while CNTF injection in the spinal cord upregulates the transcripts encoding FGF2, a potent OPC mitogen (Albrecht et al., 2003). The loss of astrocytic gp130 receptor, the ubiquitous signal transducer for CNTF, consistently exacerbates both demyelination and the proinflammatory T cell infiltration in the EAE model *via* apoptosis of astrocytes, decrease of CNS regulatory Foxp3-expressing T cells and increase of IL-17–, IFNγ– and TNF-producing T cells. The SHP2/Ras/ERK pathway was then identified as the involved intracellular mechanism (Haroon et al., 2011).

IL-1β also stimulates the astroglial production of LIF promoting survival of oligodendrocytes and thus decreasing disease severity in EAE mice (Butzkueven et al., 2002). In a pathological context where tumor necrosis factor (TNF) is a key component of the inflammatory response, LIF can alternatively be released upon TNFR2-mediated activation of the PI3K-PKB/Akt pathway in primary astrocytes. The selective stimulation of TNFR2 on astrocytes cocultured with OPCs promoted OPC differentiation into mature oligodendrocytes while the process was blocked in the presence of LIF neutralizing antibodies (Fischer et al., 2014). Reactive astrocytes also produce BDNF supporting oligodendrogenesis and regeneration after white matter damage. *In vitro*, conditioned medium from astrocytes restored the process of OPC maturation even under hypoxic stress known to block OPC differentiation unless the medium was specifically treated to remove BDNF. Similarly, *in vivo*, the conditional astroglial deletion of BDNF led to a highly reduced number of newly generated oligodendrocytes and thus to larger white matter damage in animals subjected to prolonged cerebral hypoperfusion (Miyamoto et al., 2015).

The multitude of factors secreted by astrocytes is proposed to induce a rapid change in the lesion environment sensed by OPCs, which thereby become activated. Upon demyelination, OPC activation is associated with a reversion of gene expression profile of adult OPCs towards a profile more closely resembling that of neonatal OPCs. A notable increase in the expression of genes associated with innate immune system functions (including IL1β and CCL2) was identified, as well, together with an increased ability of activated OPCs to migrate and differentiate compared with non-activated ones. Activated OPCs finally express transcription factors such as TCF4 and Sox2, which serve to maintain OPC in the cell cycle and to prime these cells for further differentiation (Moyon et al., 2015; Franklin and Ffrench-Constant, 2017).

### Pro-inflammatory Secreted Molecules Associated With Reactive Astrocytes

**Figure 2:**
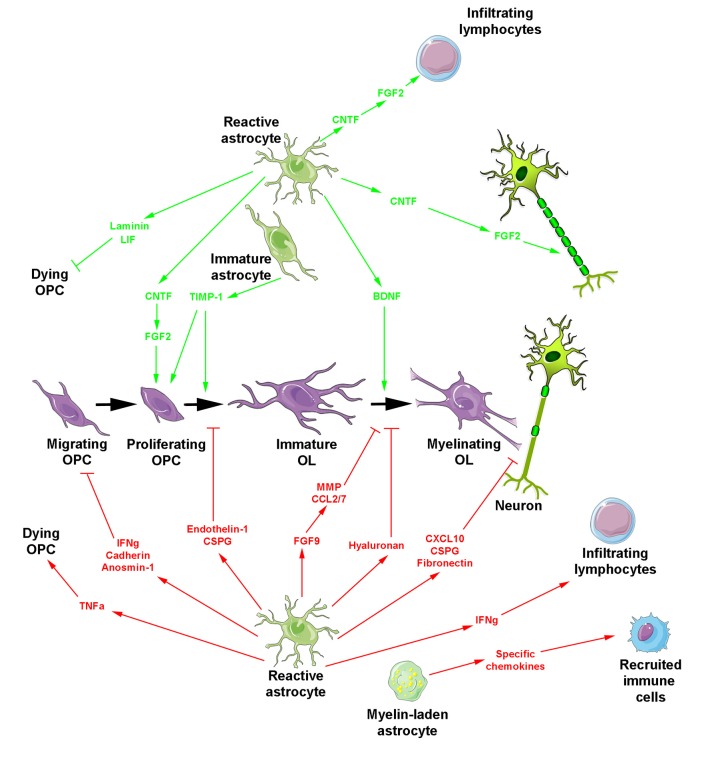
Reactive astrocyte-derived secretion products during myelin repair. The oligodendroglial lineage is shown in purple. Reactive astrocytes generate molecules promoting (top, green arrows) or blocking (bottom, red arrows and blocking symbols) myelin regeneration. Besides reactive astrocytes, two additional forms of astrocytes are indicated including myelin-laden astrocytes endowed with a deleterious effect under inflammatory conditions and transplanted immature astrocytes which promote OPC proliferation/differentiation and additionally prevent glial scar formation.

All the above activities derived from reactive astrocytes are likely involved in the spontaneous regenerative process of remyelination that can occur with remarkable efficiency, not only in experimental models of demyelination but also in humans suffering from MS (Prineas et al., 1993; Patrikios et al., 2006; Patani et al., 2007; Franklin and Ffrench-Constant, 2017). However, in chronic lesions, remyelination mostly fails or occurs only in the periphery of the plaques (Prineas and Connell, 1979; Barkhof et al., 2003; Bramow et al., 2010), where OPC migration and/or differentiation are likely impaired (de Castro et al., 2013; Franklin and Ffrench-Constant, 2017). Such failure has been associated with deleterious activities of astrocytes classically related to a more severe level of reactivity and the secretion of damaging molecules (Figure 2). One of these molecules, TNF-α, was mainly detected in fibrous astrocytes at the periphery of chronic active MS lesions and mostly transcribed in highly demyelinated plaques (Hofman et al., 1989; Selmaj et al., 1991; Bitsch et al., 2000). Although it is not yet clear whether TNF-α is internalized or produced by astrocytes (Selmaj et al., 1991; Aranguez et al., 1995), the direct physical contact between astrocytes and oligodendrocytes was proposed to be necessary for oligodendrocyte apoptosis by TNF-α in glial cell cultures. One possibility for contact-dependent cell killing is through gap junctions known to couple astrocytes and oligodendrocytes (Nagy and Rash, 2000; Orthmann-Murphy et al., 2007) and previously suggested for the propagation of cell injury (Lin et al., 1998; Farahani et al., 2005; Froger et al., 2010; Kim et al., 2011).

In the same manner, the knockout of the IFN-γ receptor in astrocytes was found to decrease chemokine expression and inflammatory cell infiltration thus preventing demyelination and consequently lowering clinical signs, namely by alleviating both Th1- and Th17-mediated adoptive EAE (Ding et al., 2015). Together with suggesting an important role for IFN-γ signaling in astrocytes during autoimmune CNS inflammation, this phenotype was consistent with the delay in disease recovery observed in transgenic EAE mice ectopically expressing IFN-γ in astrocytes during the recovery stage and also with the dramatic reduction of oligodendroglial repopulation in the demyelinated lesions of the cuprizone model upon CNS delivery of IFN-γ (Lin et al., 2006). The detection of IFN-γ in astrocytes of active chronic MS plaques and its ability to induce class II major histocompatibility antigen HLA-DR-Ia in astrocytes *in vivo* suggested that IFN-γ may play an important role in the development of MS lesions (Traugott and Lebon, 1988; Hashioka et al., 2009).

Another cytokine secreted by astrocytes is CXCL10 that is namely detectable around active MS lesions (Ransohoff et al., 1993; Omari et al., 2005; Carter et al., 2007). Its production increases before the onset of clinical symptoms, maintain at high levels during the peak of the disease and decreases during remission in the EAE model (Glabinski et al., 1997; Fife et al., 2001). *In vitro*, CXCL10 is upregulated in an astrocyte phenotype endowed with an inhibitory role in glial/axonal ensheathment without impairment of OPC proliferation/differentiation or process extension (Nash et al., 2011).

Among the other molecules highly expressed by reactive astrocytes, endothelin-1 also stands as a strong inhibitor of remyelination. This neuropeptide acts by promoting Notch activation in OPCs through induction of Jagged1 expression in reactive astrocytes (Hammond et al., 2014). In the same line, lesion formation was reported to be associated with increased expression of fibroblast growth factor 9 (FGF9) by astrocytes acting not *via* a direct effect on the oligodendroglial lineage, but *via* an off-target effect mediated by soluble astrocyte-derived factors inhibiting terminal differentiation of myelinating oligodendrocytes. *In vitro*, this activity is associated with the appearance of multi-branched “pre-myelinating” oligodendrocytes that extend processes able to interact with axons without forming any myelin sheaths, reminiscent of the oligodendrocyte phenotype observed in chronically demyelinated MS lesions. Astrocyte-derived factors included metalloproteases associated with ECM remodeling and the pro-inflammatory chemokines CCL2 and CCL7 that contribute to the development of inflammatory responses in the CNS (Lindner et al., 2015).

### Reactive Astrocytes Secrete Extracellular Matrix Deleterious for Remyelination

Apart from cytokines, growth factors and neuropeptides, ECM molecules are also secreted by reactive astrocytes and highly modify the lesion environment in MS, consequently influencing the behavior of OPCs (Maier et al., 2005; van Horssen et al., 2005, 2006; Clemente et al., 2011; Pu et al., 2018). Both the receptors present at the surface of OPCs and the ECM molecules secreted by reactive astrocytes are susceptible to change the environment from permissive to inhibitory for remyelination (de Castro et al., 2013). Indeed, except the interaction reported between astroglial laminin and oligodendroglial α6 β1 integrin shown to attenuate oligodendrocyte death *in vitro* (Corley et al., 2001), ECM accumulation appears as an important factor in tissue regeneration failure. For instance, the expression of N-cadherin on the surface of both oligodendrocytes and astrocytes led to the first *in vitro* evidence that this molecule was in part responsible for the poor migration-promoting properties induced by astrocytes on oligodendrocytes suggesting that the inhibition of cell-cell adhesion could improve repopulation of MS lesions by OPCs. Indeed, the blocking of cadherin function by specific peptides reduced adhesion of oligodendroglia to astrocyte monolayers, diminished the duration of the contact between oligodendrocyte processes and individual astrocytes, and increased the migration of oligodendrocytes on astrocyte monolayers (Schnadelbach et al., 2000). In the same line, astrocytes in culture produce fibronectin as fibril-like structures in inflammatory conditions, known to inhibit myelin formation in different experimental paradigms (Sisková et al., 2009). In agreement with this hypothesis, fibronectin rapidly accumulates as an acute response to demyelination in MS and disappears during remyelination, whereas astrocyte-released aggregated fibronectin persists in chronic lesions (Stoffels et al., 2013). Similarly, the ECM-associated glycoprotein anosmin-1 can also be detected in the core of chronic active and inactive MS plaques where it is suggested to prevent OPC colonization rather than inhibit their differentiation *via* a mechanism possibly involving the FGF-2 receptor FGFR1 consistently detected in the periplaque of chronic lesions (Bribián et al., 2006, 2008; Clemente et al., 2011).

Major CNS ECM proteoglycans have also been characterized in MS lesions. In active plaque edges, the chondroitin sulfate proteoglycans (CSPG) versican, aggrecan, neurocan and the dermatan sulfate proteoglycan increase in correlation with astrocytosis. In contrast, these molecules accumulate in foamy macrophages in active plaque centers, suggesting their engulfment together with myelin. In inactive lesions and normal-appearing white matter, proteoglycans are decreased and display undetectable abnormal heterogeneous aggregation, respectively (Sobel and Ahmed, 2001). The roles of CSPG during development in particular in myelination and the different localizations of CSPG according to the type of MS lesions both support the idea that these molecules comprising part of the astrogliotic scar, are critical in inhibition of OPC processes outgrowth and OPC differentiation (Siebert and Osterhout, 2011). The hypothesis is consistent with the recent design of a novel CSPG synthesis inhibitor reducing CSPG deposition into the lesion microenvironment and found to rescue OPC process outgrowth *in vitro* and to accelerate remyelination following focal demyelination in mice (Keough et al., 2016). Moreover, the glycosaminoglycan hyaluronan characterized by its ability to bind CSPG is deposited in early MS lesions in a low molecular weight form by lymphocytes and microglia / macrophages whereas it is deposited by astrocytes in a higher molecular weight form in chronic lesions. There, hyaluronan degraded by hyaluronidases expressed by OPCs inhibits OPC maturation and thus remyelination (Back et al., 2005) through a mechanism requiring the Toll-like receptor 2 expressed by oligodendrocytes and upregulated in MS lesions (Sloane et al., 2010). Evaluated in the Theiler’s murine encephalomyelitis model, the spatio-temporal course of ECM alterations confirmed the correlation of matrix accumulation with astrogliosis still supporting a mainly astrocytic origin of ECM deposits. The data led to propose disturbed aggregation or post-translational modifications of matrix molecules leading to impairment of their regular degradation rather than changes in their transcription level (Haist et al., 2012).

### The Dual Activity of Astrocytes in the Demyelinated Tissue

The dual activity of astrocytes described above not only reflects more or less severe levels of astrocyte reactivity, but also clearly reminds the duality that characterizes the glial scar itself, which is considered as a rearrangement of tissue structure, astrocyte proliferation, and pronounced overlap of astrocyte processes resulting in the disruption of individual astrocyte domains (Zamanian et al., 2012; Anderson et al., 2016; Adams and Gallo, 2018). While traditionally viewed as a barrier to axon regeneration, the glial scar has been nevertheless reported to be beneficial for restricting leukocyte migration outside the damaged tissue (Faulkner et al., 2004; Okada et al., 2006; Herrmann et al., 2008; Voskuhl et al., 2009). Moreover, in contrast to the initial dogma, its deleterious effect on axon regeneration was recently questioned. Indeed, genetically targeted loss-of-function approaches leading to the prevention of astrocyte scar formation, attenuation of scar-forming astrocytes or deletion of chronic astrocyte scars, failed to result in spontaneous regrowth of transected CNS axons following spinal cord injury lesions. These data were corroborated by results of RNA sequencing revealing the expression of multiple axon-growth supporting molecules namely in astrocytes (Anderson et al., 2016).

Astrocyte ability to exhibit different phenotypes participates in this heterogeneity (Zamanian et al., 2012; Liddelow and Barres, 2017). Indeed, despite a core set of genes up-regulated in different injury models such as ischemic stroke and neuroinflammation, 50% of altered gene expression is specific to a given injury. The first transcriptomic analyses provided evidence for the existence of two subtypes of reactive astrocytes (Zamanian et al., 2012) afterwards named “A1” and “A2.” A1 astrocytes induced by classically activated neuroinflammatory microglia following exposure to lipopolysaccharide have been described as harmful by inhibiting OPC proliferation and differentiation. A2 astrocytes identified during the regeneration stage in an ischemia model, were found to up-regulate genes thought to be protective. In line with this observation, analysis of MS lesions reported the detection of A1 mostly in the active lesions whereas A2 was found during remyelination (Haindl et al., 2019). However, it may be more likely that the occurrence of different astrocytic phenotypes and the existence of suitable levels of various factors expressed at particular time points may achieve successful repair. In agreement with this hypothesis, investigation of the astrocytic reaction at various steps of lesion progression in the rat EAE model led to detect simultaneously in shadow plaques all features of a glial scar and densities of OPCs and mature oligodendrocytes, which were quite comparable to the densities observed in unaffected white matter (Haindl et al., 2019).

The first line response of astrocytes to myelin injury consisting of myelin phagocytosis was similarly considered to be either beneficial or detrimental to the lesion pathology, depending on the inflammatory context. Indeed, although myelin debris phagocytosis was mainly reported to be performed by microglia/macrophages, myelin-positive hypertrophic astrocytes can be observed at sites of acute myelin breakdown. The uptake was proposed to rely on receptor-mediated endocytosis and to result in astroglial NF-kB activation and secretion of specific chemokines. The latter leads to the recruitment of immune cells shown *in vitro* in rodents and validated in human disease. Although microglia and monocyte recruitment may be beneficial for myelin clearance in a non-inflammatory environment, it increases tissue damage in demyelinating conditions driven by inflammation such as MS since additional recruitment of lymphocytes and microglia/macrophages exacerbates the inflammatory process (Ponath et al., 2017).

Finally, the finding that transplanted immature astrocytes are unable to become reactive after CNS injury (Jiang et al., 2013) and that only immature (but not mature) astrocytes are neuroprotective and suppress endogenous astrocyte activation and glial scar formation presently stands as an interesting novel concept (Chen et al., 2015). Thus, transplanted human pluripotent stem cell-derived astrocytes at a defined immature stage were shown to regulate the differentiation of endogenous OPCs, to promote myelinogenesis and to improve behavioral outcome in the model of periventricular leukomalacia in neonate mice *via* a mechanism depending on the tissue inhibitor of metalloproteinases TIMP-1 (Jiang et al., 2016).

### Key Studies Supporting Detrimental Roles of Astrocytes on Oligodendrocytes and Myelin

Several publications using astrocyte ablation have improved our knowledge about the main detrimental roles of reactive astrocytes in the context of CNS demyelination. For instance, OPC transplantation in ethidium bromide-demyelinated animals in the presence or absence of astrocytes indicated that astrocyte-free regions favor Schwann cell differentiation whereas the presence of astrocytes delayed the interaction of OPCs with the demyelinated axons (Blakemore et al., 2003). Similarly, the depletion of astrocytes *via* intracallosal injection of La-aminoadipate in cuprizone-treated animals revealed a notable increase in the percentage of myelinated areas, decrease in Iba-1^+^ microglia staining and collapse in the expression of genes related to either recruitment of microglia classically triggered by astrocytes or suppression of OPC differentiation (Madadi et al., 2019). The considerable amount of fibronectin produced by astrocytes and the ability of astrocytic fibronectin to become aggregated after treatment with lipopolysaccharide led to conclude to the deleterious effects of such aggregates on oligodendrocyte differentiation and myelin regeneration *in vivo*, in agreement with the detection of a low level of fibronectin aggregates in remyelinated MS lesions (Stoffels et al., 2013). The signaling molecule endothelin-1 (ET-1) expressed by reactive astrocytes in MS and murine demyelinated lesions is a negative regulator of OPC differentiation and remyelination acting by promoting Notch activation in OPCs during remyelination (Hammond et al., 2014).

Moreover, the CCL2 chemokine produced by astrocytes plays an important role in the continued recruitment of immune cells and the activation of glial cells in the CNS during chronic EAE (Kim et al., 2014). Similarly, the adhesion molecule VCAM-1 expressed by astrocytes is essential for T cell entry into the CNS parenchyma from EAE animals (Gimenez et al., 2004). As previously mentioned, myelin uptake is an early response of astrocytes in diseases with prominent myelin injury that results in the recruitment of immune cells possibly increasing tissue damage in demyelinating conditions driven by inflammation (Ponath et al., 2017). In the same line, astrocytes constitute one of the cell types that have the capacity to express molecules required for antigen presentation under inflammatory conditions required for T cell reactivation targeting the myelin sheath-forming oligodendrocytes (Waisman and Johann, 2018). Finally, although in the healthy brain, perivascular astrocyte end-feet closely ensheath the microvasculature, activation of perivascular astrocytes may alter their involvement in the blood-brain barrier formation as suggested by the damage of perivascular end-feet reported in MS patients. The revisit of this observation in the EAE model showed that reactive astrocytes detach from the blood vessels and lose their contacts with both blood vessels and neuronal synapses, which was proposed to contribute to the neurological impairment and the cognitive decline occurring in EAE/MS as well as to the neurodegenerative disease progression (Eilam et al., 2018).

## Microglia in Remyelination

In the context of myelin repair, microglia and their hematopoietic cell-derived counterparts, macrophages, both contribute to the regenerative process through key effects including the phagocytosis of myelin debris, the secretion of cytokines, chemokines, growth factors or soluble mediators and the recruitment/differentiation of progenitors at the lesion site. Although microglia and macrophages can now be distinguished by using a few specific markers (Bennett et al., 2018), they have been indistinguishable for a long time. Therefore, “microglia/macrophages” will be used for most presently available data.

### Microglia/Macrophage Phenotypes Upon CNS Demyelination

As resident immune cells of the CNS, microglia/macrophages become activated as soon as they detect any insult. This activation implicates changes in morphology and transcriptional profiles of the cells. The failure of microglia to convert from a “resting state” with a branched-like morphology to an “activated state” with an amoeboid-like morphology was namely observed upon knocking down either the β-galactoside-binding lectin Galectin-3 (Reichert and Rotshenker, 2019) or one of the receptors of the secreted proteins Sonic hedgehog, the protein Boc (Zakaria et al., 2019).

**Figure 3:**
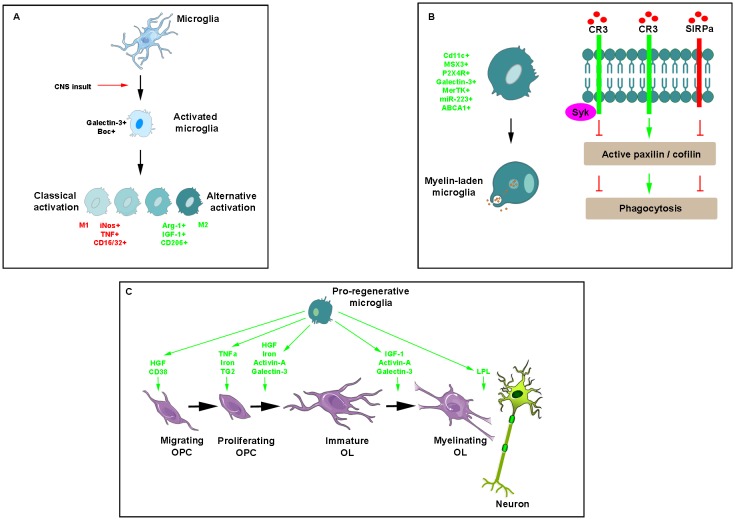
Key effects of microglia/macrophages during myelin repair.** (A)** Illustration of morphological modifications of ramified microglia/macrophages, which adopt a rhomboid shape in the presence of any insult and become activated into a classical or alternative manner giving rise to a range of cell phenotypes expressing mostly pro- (red) or anti-inflammatory (green) molecules, respectively. **(B)** Phagocytosis of myelin debris is a critical activity of microglia/macrophages for promoting myelin regeneration. Phagocytic receptors such as CR3 advance phagocytosis by promoting the activation of paxillin and cofilin whereas the immune inhibitory receptor SIRPα, inhibits phagocytosis by promoting the inactivation of the two. Recruited by CR3, Syk downregulates CR3-mediated myelin phagocytosis protecting phagocytes from excessive phagocytosis during prolonged exposure to debris. **(C)** Pro-regenerative microglia secrete a range of molecules intervening at various steps of myelin repair.

Like astrocytes, microglia/macrophages are endowed with a dual activity supported by the existence of different phenotypes observed under various *in vitro* conditions and that have been named, respectively the classical activated state (M1) associated with pro-inflammatory activities and the alternative polarized state (M2) involved in OPC recruitment and differentiation associated with anti-inflammatory activities (Figure 3A). The characterization of these phenotypes performed in the model of focal demyelination induced by stereotaxic injection of lysolecithin into the corpus callosum led to the identification of a few specific markers namely including iNos, TNF, CD16/CD32 for the classical activated state and Arg1, IGF1 and CD206 for the alternative state. The switch from an M1- to an M2-dominant response was detected in microglia/macrophages at the initiation of remyelination (Miron et al., 2013). It was more recently characterized as a process requiring necroptosis of pro-inflammatory microglia and subsequent repopulation to a pro-regenerative state (Lloyd et al., 2019). *In vitro* experiments revealed the ability of M2 cell conditioned media to promote OPC differentiation whereas the depletion of M2 cells in focal demyelinating lesions prevented OPC differentiation. Also, parabiosis experiments detected a higher number of M2 cells in lesions of aged mice in which remyelination was increased by coupling to a younger mouse. Finally, the blockade of activin-A secreted by M2 cells fully prevented OPC differentiation in demyelinated cerebellar slice cultures (Miron et al., 2013).

Microglia/macrophage activation cannot be nevertheless regarded as a simple dichotomy of M1 or M2, but undoubtedly as a much broader reactive phenotype spectrum. In agreement with this observation, in the Theiler’s induced demyelinating disease model, the endogenous cannabinoid 2-AG induces pro-phagocytic response of microglia and OPC differentiation, without any detectable regulation of the main markers used to identify classic and alternatively activated microglia, respectively (Mecha et al., 2019). In the same line, myelin debris was consistently proposed to promote the emergence of a novel phenotype that differs from conventional M1 and M2. Indeed, myelin-induced macrophages appear foamy, displaying a striking deficiency of the expression of the ATP-binding cassette transporter ABCA1 required for lipid efflux in response to myelin debris loading, thus suggesting that this homeostatic mechanism characterizing non-myelin-induced macrophages is overwhelmed. The impaired capacity of foamy macrophages for apoptotic and necrotic cell clearance is consistent with this hypothesis and likely contributes to the development of secondary injury (Wang X. et al., 2015).

In agreement with this observation, a growing number of recent publications support the existence of a wide spectrum of microglia activation states evidenced by single-cell and bulk RNA sequencing approaches characterizing this process not as a simple phenotypical change but as a dynamic response involving spatially and transcriptionally distinct subpopulations. High-dimensional single-cell mapping in EAE mouse CNS led to a unique activation profile comprising universal markers of microglia/macrophage activation including CD44, CD86, programmed death-ligand 1 (PDL1) as well as a decreased CD14 expression and increased major histocompatibility complex II (MHC II) and stem cell antigen-1 (Sca-1) expressions notably not detected in old mice or Alzheimer’s disease models (Mrdjen et al., 2018). Single-cell RNA sequencing of microglia from LPC-mediated lesions revealed multiple gene expression profile clusters of activated microglia/macrophages sharing common genes such as apoE and unique genes such as Ccl4 or Cxcl10 according to the clusters (Hammond et al., 2019). Multiple activation states specifically reflecting microglia (and not macrophages) was also identified upon LPC-mediated demyelination suggesting that microglia can have multiple forms of activation likely identified by common or selective transcriptional programs (Plemel et al., 2020). Similarly, a specific ApoE-dependent molecular signature was identified in microglia/macrophages from different models of neurodegenerative diseases including EAE (Krasemann et al., 2017). Strikingly, one of these subsets displayed an expression profile comparable to the one found in MS microglia (Zrzavy et al., 2017). Real-time *in vivo* imaging of microglia/macrophages in EAE mice visualized the expected switch from pro-inflammatory to immuno-regulatory markers mostly in initial lesions with an increase during lesion resolution (Locatelli et al., 2018). Consistently, in actively demyelinating lesions from MS patients, some microglia/macrophages express pro-inflammatory markers while active lesions prone to re-myelinate comprise microglia/macrophages expressing anti-inflammatory markers at a higher level than chronic inactive lesions (Miron et al., 2013; Zrzavy et al., 2017).

### Factors Controlling the Pro-regenerative Microglia/Macrophages

Recently, the subpopulation of CD11c-expressing microglia previously reported to be transiently expanded soon after birth and critical for primary myelination, was found to be remarkably increased in EAE and cuprizone models of demyelination. The expansion of this subpopulation is induced by the stimulation of CSF1R, the key transducer of CSF1 and interleukin (IL)-34 signals, essential in myeloid cell development. CSF1R activation induces the expression of the chemokine CCL2, itself leading to a dramatic increase of CD11c^+^ microglia, finally resulting in improvement of EAE symptoms and lower levels of demyelination (Wlodarczyk et al., 2018). Among factors implicated in the promotion of the remyelinating phenotype of microglia, the homeobox gene Msh-like homeobox-3 (MSX3) is a pivotal regulator for microglial polarization that is induced and repressed in M2 and M1 cells, respectively. This finding is supported by the dynamic regulation of MSX3 in the EAE model as well as by the beneficial and deleterious effects observed after MSX3 gain- and loss-of-function experiments in different demyelination models. Pparg, Stat6, and Jak3 are key genes regulated by MSX3 (Yu et al., 2015). In the same line, pro-regenerative microglia express a high level of the purinergic receptor P2X4R at the peak of recovery in the EAE model. P2X4R activation increases and decreases pro-regenerative and pro-inflammatory genes, respectively, whereas its blockade prevents myelin debris clearance (Zabala et al., 2018).

### The Critical Role of Microglia/Macrophages in Myelin Phagocytosis

The ability of microglia/macrophages to phagocyte myelin debris is critical for promoting OPC recruitment and differentiation into myelinating cells (Triarhou and Herndon, 1985; Kotter et al., 2001, 2005, 2006; Neumann et al., 2009). The main phagocytic receptor in microglia/macrophages is the complement receptor-3 (CR3). Binding of myelin to CR3 initiates structural changes characteristic of phagocytosis mostly reflected by filopodia-like membrane protrusions that engulf myelin as they extend and then pull myelin into phagocytes as they retract. Filopodia production depends on F-actin remodeling, which is promoted by active unphosphorylated cofilin and impeded by inactive phosphorylated cofilin. Spleen tyrosine kinase (Syk), the non-receptor tyrosine kinase known to be recruited by phagocytic receptors including CR3, downregulates CR3-mediated myelin phagocytosis by increasing the inactive state of cofilin. This self-negative control of phagocytosis is useful in protecting phagocytes from excessive phagocytosis during prolonged exposure to particles that are fated to ingestion (Hadas et al., 2012). Remarkably, myelin debris can inhibit its clearance by using a protective mechanism whereby CD47 protein expressed by myelin debris binds the immune inhibitory receptor signal regulatory protein-α (SIRPα) on the surface of phagocytes (Figure 3B). The mechanism supporting such detrimental protection involves the regulators of cytoskeleton function paxillin and cofilin, which are both either activated by phagocytic receptors or inactivated by the immune inhibitory SIRPα (Gitik et al., 2014). The finding that expression of Galectin-3 correlates with myelin-debris phagocytosis in microglia led to show its implication in phagocytosis activation first by advancing cofilin activation, causing filopodia/lamellipodia to extend/engulf myelin-debris and, second, by advancing actin/myosin-based contraction through K-Ras.GTP/PI3K signaling, causing filopodia/lamellipodia to retract and thus internalize myelin debris (Reichert and Rotshenker, 2019).

The retinoid X receptor (RXR) plays an important role in monocyte/macrophage phagocytosis of myelin as suggested by pioglitazone-induced activation of peroxisome proliferator-activated receptor c, one of the permissive binding partners of RXR, shown to inhibit pro-inflammatory differentiation of MS patient-derived monocytes/macrophages and to enhance myelin phagocytosis (Natrajan et al., 2015b). Moreover, the blockade of myelin debris clearance by microglia/macrophages in cuprizone-demyelinated CX3CR1-deficient mice, revealed the critical role played by CX3CR1, the receptor controlling microglial physiology and orchestrating the crosstalk between microglia and neurons (Lampron et al., 2015). Moreover, macrophages lacking miR-223 expression have impaired ability to polarize towards the M2 phenotype thus altering myelin debris clearance and remyelination in the lysolecithin-demyelinated corpus callosum (Galloway et al., 2019). Interestingly, the use of human monocyte-derived macrophages and microglia led to demonstrate that myelin phagocytosis is significantly enhanced in cells exposed to TGF-β compared with resting basal conditions whereas it is markedly reduced in classically activated polarized cells. The transcriptional analysis of TGF-β–treated microglia revealed the tyrosine kinase receptor MerTK as one of the most upregulated among differentially expressed genes. In contrast, the enzyme and its ligands (growth arrest-specific 6 and Protein S) are down-regulated in classically activated cells (Healy et al., 2016).

Recent publications identified novel signaling pathways aimed at restoring homeostatic microglial phagocytosis in the aging CNS. Indeed, old mice fail to resolve the inflammatory response initiated after myelin damage. This observation was related to the accumulation of excessive amounts of myelin debris by aged phagocytes, which trigger cholesterol crystal formation and phagolysosomal membrane rupture, thus stimulating inflammasomes. However, stimulation of cholesterol efflux and solubility by administering 2-hydroxypropyl-β-cyclodextrin in old demyelinated mice knockout for the major cholesterol transporter ApoE was sufficient to restore the capacity to re-myelinate lesioned tissue (Cantuti-Castelvetri et al., 2018).

Combined CRISPR-Cas9 knockout screens together with RNA-seq led to discover age-related genetic modifiers of microglial phagocytosis such as the canonical B-cell receptor CD22, a negative regulator of phagocytosis that is upregulated on aged microglia. CD22 mediates the anti-phagocytic effect of α2–6-linked sialic acid. Its inhibition promotes the clearance of myelin debris *in vivo* and reprograms microglia towards a homeostatic transcriptional state leading to improvement of cognitive function in aged mice (Pluvinage et al., 2019). By up-regulating the expression of the scavenger receptor CD36, niacin (also called vitamin B3) induces myelin phagocytosis through binding its receptor, hydroxycarboxylic acid receptor 2, both *in vitro* and *in vivo* (Rawji et al., 2020).

### Pro-regenerative Microglia/Macrophage-Derived Factors

Many cytokines, growth factors, and soluble factors are known to be secreted by microglia/macrophages (Figure 3C). Although induced by IL1β characterized as a pro-inflammatory cytokine (Mason et al., 2001), microglia/macrophage-derived IGF-1 enhances remyelination as suggested by EAE rats receiving IGF-1 subcutaneously (Yao et al., 1996) and in lysolecithin-demyelinated spinal cord of young and aged rats intrathecally infused with the growth factor (Hlavica et al., 2017). TNFα through its TNFR2 receptor promotes remyelination by increasing OPC proliferation as shown by using mouse strains knockout for the ligand or its receptor as well as by their up-regulation during the remyelination stage in the cuprizone model (Arnett et al., 2001). T helper cell-derived TGF-β induces the secretion of microglial hepatocyte growth factor (HGF), which drives OPC chemotaxis and differentiation. In agreement with this finding, spinal cord lesions from relapsing-remitting EAE mice contain both OPC and HGF-producing microglia/macrophages in the recovery-, but not in the acute phase (Lalive et al., 2005). Highly expressed in microglia, the β-galactoside-binding lectin, galectin-3, has a critical role in driving OPC differentiation and myelination in agreement with the oligodendrocyte maturation effects displayed by supernatants derived from galectin-3-expressing- but not galectin-3-deficient microglia (Pasquini et al., 2011).

Microglia/macrophages also express activin-A at onset of remyelination of focally-induced demyelination. This growth factor regulates oligodendrocyte differentiation and myelin compaction *via* the activin receptor subtype Acvr2a up-regulated during efficient myelin regeneration, in contrast to the other receptor subtype Acvr2b, which has to be down-regulated for allowing oligodendrocyte differentiation. In actively remyelinating areas of MS tissue, oligodendrocyte lineage cells expressing Acvr2a consistently outnumber cells expressing Acvr2b suggesting that an increase in Acvr2b expression may impair oligodendrocyte differentiation and myelin formation induced by Acvr2a (Dillenburg et al., 2018).

Finally, microglia is the main iron source in the form of ferritin. The latter is required for OPC proliferation and differentiation and stands as an essential cofactor for enzymes involved in myelin cholesterol and fatty acid synthesis (Schonberg et al., 2012). The cellular iron level in microglia/macrophages is controlled by hepcidin and ferroportin. The transcription of the former is regulated by the BMP/Smad and IL-6/Jak-STAT3 signaling pathways during inflammation resulting in the degradation of the only known iron exporter ferroportin, subsequently increasing intracellular iron level. Recently, the infusion of the BMP antagonist noggin in a model of ischemic stroke decreased the induction of hepcidin and ferritin proteins whereas it increased the number of myelinated axons and myelin thickness (Shin et al., 2018).

Several microglial enzymes are also clearly implicated in remyelination. As shown in development, microglia-derived TG2 signals to GPR56 on OPCs in the presence of the ECM protein laminin promote OPC proliferation and improves remyelination *in vivo* in the cuprizone model and *ex vivo* in cerebellar slices exposed to lysolecithin (Giera et al., 2015). Lipoprotein-lipase involved in lipid-processing, which plays an important role during initiation of remyelination and is considered as a feature of alternatively-activated microglia, is significantly increased in the EAE model when clinical symptoms start to decrease (Bruce et al., 2018; Kamermans et al., 2019). Finally, CD38 that catalyzes the synthesis of cyclic adenosine diphosphate-ribose (cADPR) from nicotinamide adenine dinucleotide (NAD^+^), is increased in the cuprizone model of demyelination in both astrocytes and microglia and may be required for myelin clearance and oligodendrocyte repopulation (Roboon et al., 2019).

Recently, extracellular vesicles produced *in vitro* by either pro-inflammatory- or pro-regenerative microglia were proposed to be one of the mechanisms used to promote ou block myelin repair. Remarkably, exposure of cultured OPCs to inflammatory vesicles blocked OPC maturation only in the presence of astrocytes, thus implicating the latter in remyelination failure *via* a mechanism involving astrocyte conversion into deleterious cells. Moreover, biochemical fractionation revealed that the inflammatory cargo of the pro-inflammatory vesicles mainly contribute to the blockade of OPC maturation whereas surface lipid components may be mostly involved in extracellular vesicle-mediated migration and/or differentiation of OPCs likely implicating sphingosine 1 phosphate at least for OPC migration (Lombardi et al., 2019).

### Therapeutic Modulation of Microglia/Macrophages

Major improvement of our knowledge of the role of microglia/macrophages during remyelination recently led to new therapeutic perspectives. One of the first papers providing evidence that increased recruitment of microglia/macrophages can support remyelination, regarded the anti-fungal amphotericin B and its association with macrophage colony-stimulating factor (M-CSF). Although using amphotericin B is precluded by its ability to stimulate TNF secretion and its toxic properties (Doring et al., 2015), the increased uptake of myelin debris induced by M-CSF in the cuprizone model remains interesting (Laflamme et al., 2018). The adoptive transfer of bone-marrow-derived M2 macrophages results in a shift of the immunological response from helper T1 to helper T2 lymphocytes through the production of anti-inflammatory cytokines, which in turn induces the polarization of microglia/macrophages to the M2 phenotype in a model of spinal cord injury (Ma et al., 2015). Preventive IL-13 gene therapy in the cuprizone model directs the polarization of microglia and infiltrating macrophages towards an alternatively activated phenotype, thereby limiting lesion severity and improving disease outcome (Guglielmetti et al., 2016).

Several molecules modulating microglia/macrophages are presently trialed in patients. For instance, the anti-muscarinic molecule Clemastine, modulates macrophage inflammatory responses *in vitro* and microglia/macrophage activation *in vivo* (Mei et al., 2014). Bexarotene, an agonist of the RXR pathway, has been identified as a positive regulator of myelin debris clearance and a key player in the age-related decline in remyelination acting *via* a yet undetermined direct or indirect effect (Natrajan et al., 2015a). The antipsychotic quetiapine fumarate promotes remyelination *in vivo* together with attenuating microglial responses to inflammatory stimuli by decreasing TNF and nitric oxide secretion and preventing activation of NF-κB (Wang H. et al., 2015).

New molecules are continuously being identified. Among them, a recombinant version of a naturally occurring human IgM, rHIgM22, has been shown to promote remyelination in Theiler’s virus infection and cuprizone animal models and to stimulate myelin phagocytosis in a dose-dependent manner (Zorina et al., 2018). The CSF1R kinase inhibitor BLZ945 prophylactically and therapeutically prevents demyelination in the cuprizone model, namely in the corpus callosum. However, it increases myelin debris and axonal damage in other fiber tracts reminding the phenotype observed in cuprizone-treated TREM2 knock-out mice (Beckmann et al., 2018; Wies Mancini et al., 2019). Although TNF is a master pro-inflammatory product of activated microglia/macrophages implicated in CNS demyelination, the blockade of its soluble form did not prevent cuprizone-induced oligodendrocyte loss and demyelination, but led to efficient early remyelination due to improved phagocytosis of myelin debris and resolution of microglia/macrophage activation (Karamita et al., 2017). Finally, the allosteric modulator of P2X4R, Ivermectin, mimics P2X4R activation and improved motor function, electrical nerve conductance, myelin debris phagocytosis and remyelination in the EAE model (Zabala et al., 2018).

### Key Studies Supporting Detrimental Roles of Microglia/Macrophages on Oligodendrocytes and Myelin

Besides the growing number of data supporting the beneficial consequences of promoting the pro-regenerative phenotype of microglia/macrophages, several critical works support the deleterious effects of microglia activated through the classical way. First, activated microglia synthesize a multitude of cytokines, chemokines, cell adhesion glycoproteins or reactive oxygen radicals able to damage axons, myelin, oligodendrocytes (Minghetti and Levi, 1998; Raivich and Banati, 2004) and therefore involved in the initiation and propagation of the inflammatory cascade, which promotes demyelination in neural disorders (Perry et al., 2010). Consistently, LPS-activated microglia, polarized to pro-inflammatory status secrete tumor necrosis factor-α (TNFα) and interleukin-1β (IL-1β), both known to be cytotoxic for oligodendrocytes (Selmaj and Raine, 1988). TNFα and cuprizone supplementation to rat primary cultures of oligodendrocytes consistently decreased cell viability (Pasquini et al., 2007). Moreover, a minocycline-mediated blockade of microglial activation in cuprizone-treated mice prevented demyelination while a positive correlation was shown between the production of nitric oxide and oligodendrocyte death (Merrill et al., 1993). Finally, LPS-induced secretion of the stress chaperone protein, heat shock protein 60 (HSP60), was found to initiate OPC apoptosis (Li et al., 2017).

As competent presenters of antigen, microglia activated in a classical way express molecules for antigen presentation such as MHC II and its co-stimulatory factors CD40 and CD86, characterized as classical markers of microglia activation in MS and Alzheimer’s disease patients (Gobin et al., 2001; Lehmann et al., 2001; Höftberger et al., 2004). Remarkably, activated microglia/macrophage is also highly proliferative in both MS and animal models of demyelination mostly in actively demyelinating areas at the early stages of the disease but not in remyelinating lesions again attesting the major role of classically activated microglia/macrophages during the early stages of demyelinating diseases (Matsumoto et al., 1992; Schönrock et al., 1998; Ponomarev et al., 2005). Early activation of microglia is similarly observed in ischemic dementia models as shown by the increased expression of MHC-I/II or matrix metalloprotease-2 (MMP-2) 3 days after hypoperfusion whereas administration of the natural dipeptide carnosine (β-alanyl-L-histidine) able to decrease microglial activation improved cognitive function and white matter lesions (Wakita et al., 1994; Ihara et al., 2001). Finally, as previously mentioned, extracellular vesicles released by pro-inflammatory microglia block remyelination *via* a mechanism depending on the presence of astrocytes converted into harmful cells by the inflammatory extracellular vesicle cargo (Lombardi et al., 2019).

In summary, the majority of experimental evidence reported in this review, points to astrocytes and microglia/macrophages as being crucial in both developmental and repairing oligodendrogenesis and myelination. During development, astrocytes and a unique phenotype of neonatal microglia secrete different patterns of molecules controlling oligodendrocyte development and myelination. Although both cell types shape the number of OPCs with consequences for subsequent myelinogenesis, astrocytes are also implicated in a tight crosstalk between OPCs and axons making them indispensable for myelination. Also, by providing a substantial fraction of lipids incorporated into CNS myelin, astrocytes are required for the proper generation of the myelin sheath. Under pathological and demyelinating conditions, reactive astrocytes and activated microglia/macrophages both exhibit a dual activity inducing detrimental or beneficial effects, the balance of which appears to be critical for tissue regeneration. Besides the secretion of a wide range of molecules, both cell types can phagocyte myelin debris. However, in contrast to the beneficial role of microglia/macrophages-induced phagocytosis, myelin uptake occurring as an early response of astrocytes results in recruitment of immune cells possibly increasing tissue damage in demyelinating conditions driven by inflammation. As a whole, the review indicate that beyond the secretion of a multitude of cues directly controlling diverse facets of OPC biology, astrocytes and microglia/macrophages strongly modify the lesion microenvironment. Further delineation of this crosstalk should open the way towards novel therapeutic approaches aimed at recovering proper developmental myelination and relieving the obstacles on the failure of regeneration of damaged myelin characterizing CNS demyelinating diseases.

## Author Contributions

All authors contributed to the design and documentation of this review article.

## Conflict of Interest

The authors declare that the research was conducted in the absence of any commercial or financial relationships that could be construed as a potential conflict of interest.
